# Graft Detachment After Descemet's Stripping Automated Endothelial Keratoplasty in Bullous Keratopathy and Fuchs Dystrophy

**Published:** 2019-12-01

**Authors:** Nicola Cardascia, Valentina Pastore, Vito Bini, Maria Gabriella Lategola, Giovanni Alessio

**Affiliations:** 1 Department of Medical Science, Neuroscience and Sense Organs, Eye Clinic, University of Bari “A. Moro”,Bari, Italy

**Keywords:** DSAEK, Graft Detachment, Air Re-bubbling, Bullous Keratopathy, Fuchs Dystrophy

## Abstract

Descemet’s stripping automated endothelial keratoplasty (DSAEK) is a surgical technique for corneal transplantation in case of corneal decompensation. One of the main complications is graft detachment (GD) recoverable with Air Re-bubbling (ARB). The aim of this retrospective, interventional case series was to identify factors related to this complication in eyes operated for bullous keratopathy (BK) and Fuchs dystrophy (FD). We considered one-hundred patients who underwent DSAEK for BK or FD between January 2016 and October 2017 at Department of Ophthalmology, Policlinico Universitario of Bari, Italy. Studied parameters included physiological and pathological anamnesis of both donors and recipients and properties of donor’s lenticules and of the recipient’s corneas. Data was analyzed using One-way ANOVA with Tukey post hoc test and Chi-square test with Odds Ratio (OR) calculation. We grouped patients according to diagnosis. GD occurred in 9 eyes affected by BK and 19 by FD (p=0.003, OR = 0.25, 95% CI, 0.098-0.62). It was recovered with ARB. In BK, ARB correlated to complicated cataract extraction (p=0.04, OR = 7.83, 95% CI, 1.28 – 47.98) and aphakia (p=0.026, OR = 54.38, 95% CI, 2.51 - 11.76). In FD, ARB was associated to donor’s death for neoplasia (p=0.06, OR= 4.04, 95% CI, 1.06 – 15.37). No other differences were found. In conclusion, we could hypothesize that in FD patients, donor’s cancer therapy may play a role on altered corneal fibroblast metabolism, activating a synergetic effect between chemotherapy and genetic alteration of FD, which may lead to an altered adhesion of donor’s lenticule on recipient's stroma. In BK patients, complicated cataract extraction and aphakic status of recipients’ eye may contribute to altered adhesion of donor’s lenticule post-DSAEK.

## INTRODUCTION

Bullous keratopathy (BK) and Fuchs dystrophy (FD) are among the most common causes of corneal decompensation, often requiring corneal transplantation [1]. BK is a complication of many types of eye surgery, especially cataract extraction. It is characterized by corneal edema and stromal bubbles which cause pain and visual impairment [2]. Fuchs dystrophy is a bilateral corneal disease [3] due to an altered expression of the corneal endothelium-specific type VIII collagen [4, 5], which leads to a progressive deterioration of the endothelium. In earlier stages, it is characterized by ‘guttae’, anatomic alteration of the Descemet’s membrane, visible as dark areas under the specular microscope. In the following stages, the confluence of the guttae, progressive endothelial cell loss and impairment of their stromal drainage lead to stromal and epithelial edema with micro- and macrobubbles, associated with decreased visual acuity and pain [2, 3, 5].

In both cases, the gold standard of treatment is Descemet's stripping automated endothelial keratoplasty (DSAEK) [6-8]. Surgical complications occur in about 14% of cases and the most common one is graft detachment (GD), which occurs within the first days or up to six postoperative weeks [9, 10]. GD needs additional surgical treatment with surgical related risks. Air Re-Bubbling (ARB) is usually performed by inflating the anterior chamber with air to reattach corneal flap to the recipient's stroma [11].

In this study we aimed to identify factors related to GD after DSAEK in eyes operated for BK and FD, focusing on donors and recipient’s corneas properties and their medical history.

## METHODS

In 2017, ethical committee approval for retrospective studies was not required by Policlinico Universitario of Bari. In our Department level, a generic scientific informed consent was obtained from all patients before surgery. This study was performed in compliance with the principles of the Declaration of Helsinki.

We made a retrospective analysis of 59 eyes affected by BK and 45 eyes with FD undergoing DSAEK between January 2016 and October 2017 at the Eye Clinic, Department of Ophthalmology of the University General Hospital of Bari, Italy. Patients were identified by searching the Cornea Service of Bari Eye Clinic database. 

Data was collected including patient’s gender and age, corneal decompensation period, presence of pseudophakia, other ocular pathologies, keratometry (steep axis and average keratometry), pachymetry and possible concurrent phacoemulsification. Moreover, we analyzed data about thickness, endothelial cell density and diameter of the corneal lenticule as well as eye bank origin, donor’s gender and age, death cause and time span between death and corneal explantation. Finally, the occurrence of ARB, potentially repeated, was recorded. DSAEK was performed according to the standardized techniques [12], namely making a small corneal incision, stripping the Descemet’s membrane and endothelium layer, injecting the donor’s lenticule and positioning it with an air bubble in the anterior chamber. Pupillary block was prevented thanks to an inferior iridectomy. Then the patient was instructed to lay in a supine position in the following four hours. Anterior segment biomicroscopy and anterior segment optical coherence tomography (AS-OCT) were performed 1.5 and 12 days after the operation. AS-OCT (MS-39, CSO srl, Scandicci (FI), Italy) investigated the presence of fluid between the donor’s lenticule and patient’s stroma by means of two-dimension 24 radial lines centered on corneal apex (16 millimeters [mm] length) [13].

**Figure 1:**
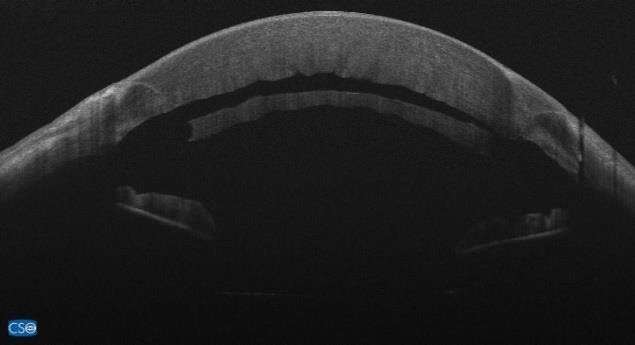
Graft Detachment at 1.5 days after Descemet's Stripping Automated Endothelial Keratoplasty. Anterior segment optical coherence tomography (ASOCT) (MS-39, CSO srl, Scandicci (FI), Italy)

**Figure 2:**
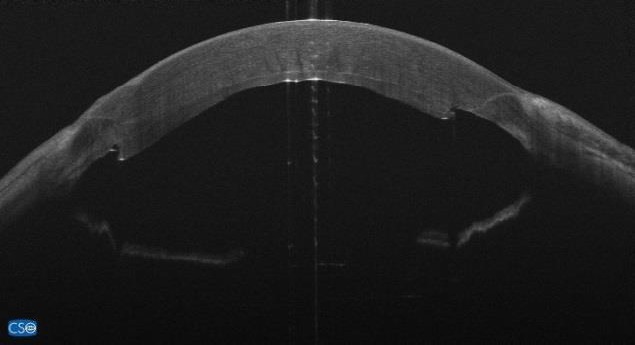
Graft repositioning with ARB at 1.5 days after Descemet's Stripping Automated Endothelial Keratoplasty. Anterior segment optical coherence tomography (ASOCT) (MS-39, CSO srl, Scandicci (FI), Italy)

**Figure 3:**
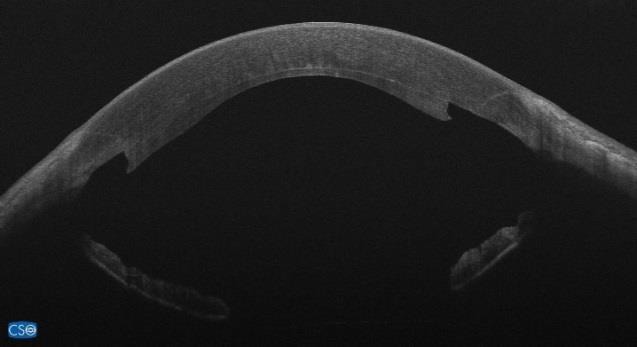
Anterior segment optical coherence tomography (ASOCT; MS-39, CSO srl, Scandicci (FI), Italy) at 12 days after Air rebubbling for management of post Descemet's Stripping Automated Endothelial Keratoplasty graft detachment.

GD was characterized by the presence of fluid between the transplanted lenticule and the recipient cornea (Figure 1). It was immediately treated by introducing an air bubble in the anterior chamber called ARB and spreading out the lenticule to attach it to the recipient’s underlying layers (Figures. 2 and 3).

DSAEK was associated to cataract extraction and posterior chamber Intra-Ocular Lens (PC-IOL) implantation in 67.8% eyes affected by FD, while it was associated with anterior chamber IOL (AC-IOL) explant and Retropupillary Iris fixated Phakic IOL implant (Verisyse, J&J Vision, USA) in 8.5% of eyes affected by BK.

Data was analyzed by One-way ANOVA with Tukey post hoc test (p<0.05) and Chi-square test with Odds Ratio calculation, GraphPad InStat (GraphPAd Software Inc. San Diego, The USA).

## RESULTS

According to corneal pathology, we categorized patients in two groups: BK and FD. BK group included 59 patients, 59 eyes and FD group 41 patients, 45 eyes.

Demographic information of patients in the both groups are resumed in Table 1. The groups were comparable regarding patient age, gender distribution and the time between diagnosis of corneal decompensation and surgery. The mean ages ± standard deviation (SD) were 68.86±11.63 years (range, 38 to 87 years) in BK and 70.51±9.08 years (range, 47 to 86 years) in FD (p=0.43, 95% CI, -2.52 – 5.81). The gender distributions (male/female) were 29/30 in BK and 24/21 in FD (p=0.82, OR= 0.85, 95% CI, 0.39 – 1.8). The mean corneal decompensation period ± SD were 11.92±14.79 months (range: 1-84) in BK and 14.11±13.35 months (range: 2-72) in FD (p=0.49, 95% CI, -3.65 – 7.59).

All eyes underwent corneal topography and pachymetry, preoperatively (Sirius CSO, Scandicci (FI), Italy). Topographic data are summarized in Table 2.

**Table 1:** Demographic Information of Eyes Affected by Bullous Keratopathy and Corneal Fuchs Dystrophy.

	Bullous Keratopathy	Fuchs Dystrophy
Eyes	59	45
Age (Y); Mean ± SD, range	68.86±11.63, 38-87	70.51±9.08, 47-86
Male/Female	29/30	24/21
Corneal decompensation period (months); mean ±SD, range)	11.92±14.79, 1-84	14.11±13.35, 2-72

Abbreviations: SD: standard deviation; Y: years.

**Table 2:** Topographic Data of Both Study Groups; Bullous Keratopathy and Corneal Fuchs Dystrophy, Preoperatively.

Recipients data: mean ± SD, (range)	Bullous Keratopathy (n=59)	Fuchs dystrophy (n=45)	P (95% CI, range)
Mean K: D	43.47±2.73 (33.99-48.74)	44.01±2.38 (40.86-55.86)	0.29 (-0.48 – 1.56)
Max K: D	45.49±2.77 (41.61-54.53)	44.95±2.59 (41.41-58.24)	0.31 (-1.06 – 0.51)
White to White: mm	11.63±0.53 (10.18-12.8)	11.59±0.44 (10.5-12.9)	0.74 (-0.23 – 0.16)
CCT: µm	838.15±192.08 (511-1388)	691.24±99.53 (545-1201)	**<0.0001** (-209.29– 84.53)

Abbreviations: SD: standard deviation; D: diopter; n: number; P: p-value; CI: confidence interval; Max: maximum; K: Keratometry reading; mm: millimeter; CCT: Central Corneal Thickness; µm: micrometer. P-value<0.05 is bold.

**Table 3:** Clinical Data Analysis of Bullous Keratopathy Versus Fuchs Dystrophy.

Ocular comorbidities	OR	95% CI	P
Viral or bacterial keratitis	11.03	30.7-39.65	**<0.0001**
Corneal edema induced by lens phacoemulsification	383.78	21.96–6707.3	**<0.0001**
Surgical aphakia	5.64	0.28-112.05	**0.02**
Complicated cataract surgery	11.06	0.61-201.78	**0.03**

Abbreviations: OR: odds ration; CI: confidence interval; P: p-value. P-value<0.05 is bold.

As shown in Table 2, the two groups were comparable in mean corneal power (Mean K), maximum corneal power (Max K) and White-to-White diameter. The Mean K ± SD were 43.47±2.73 D (range: 33.99-48.74) in BK and 44.01±2.38 D (range 40.86-55.86) in FD (p=0.29, 95% CI, -0.48 – 1.56). The Max K ± SD were 45.49±2.77 D (range 41.61-54.53) in BK and 44.95±2.59 D (range 41.41-58.24) in FD, (p=0.31, 95% CI, -1.06 – 0.51). White-to-White diameters ± SD were 11.63±0.53 mm (range: 10.18-12.8) in BK and 11.59±0.44 mm (range: 10.5-12.9) in FD (p=0.74, 95% CI, -0.23 – 0.16). A significant difference was found in recipients’ central corneal thickness (CCT): BK corneas were thicker than FD ones. The mean CCT ± SD were 838.15±192.08 µm (range: 511-1388) in BK and 691.24±99.53 µm (range: 545-1201) in FD (p<0.0001, 95% CI, -209.29 – 84.53). Clinical data are resumed in Table 3. 

There was a significant stronger association of BK, rather than FD, with ocular comorbidities such as viral or bacterial keratitis (p<0.0001, OR=11.03, 95% CI, 30.7–39.65), hard-to-overcome corneal edema induced by lens phacoemulsification (p<0.0001, OR=383.78, 95% CI, 21.96–6707.3), surgical aphakia (p=0.02, OR=5.64, 95% CI, 0.28–112.05) and complicated cataract surgery (p=0.03, OR=11.06, 95% CI, 0.61–201.78). DSAEK was combined to lens phacoemulsification and PCIOL implant in all eyes affected by FD and only in 13 eyes affected by BK (p<0.0001, OR=0.03, 95% CI, 0.01–0.11). BK was related to AC-IOL, consequent to complicated cataract extraction in 5 eyes. It was treated with AC-IOL extraction, retropupillary iris-fixated Phakic IOL (Verisyse, J&J Vision, The USA) implant and DSAEK.

Furthermore, we considered donors’ corneal parameters. The two groups were comparable in donor's whole corneal-stromal diameter, corneal thickness, endothelial cell density and diameter of implanted lenticule. The mean whole corneal-stromal diameter ± SD were 9.87±0.84 mm (range: 9-11) in BK and 9.82±0.83 mm (range: 9-11) in FD (p=0.41, 95% CI, -17.44 – 7.2). The mean corneal thickness ± SD were 99.46±36.32 µm (range: 43-285) in BK and 104.58±23.37µm (range: 62-154) in FD (p=0.86 (95% CI-49.11 – 41.05). The mean endothelial cell density ± SD were 2608±111.86 cell/mm2 (range: 2300-2800) in BK and 2604±118.62 cell/mm2 (range: 2300-2800) in FD (p=0.74, 95% CI, -0.38–0.27). The mean diameter of implanted lenticule ± SD were 8.05±0.34 mm (range: 6.5-8.5) in BK and 8.07±0.2 mm (range: 7.5-8.25) in FD (p=0.78, 95% CI, -0.97 – 0.13).

BK eyes received younger corneas than those implanted in FD. The mean donor age ± SD were 65.68±8.66 years (range: 42-79) in BK and 61.69±10.26 years (range: 36-79) in FD (p=0.03, 95% CI, -0.3–7.67). The two groups were comparable in donors’ fatal disorders ratio. We found vascular disorders BK/FD= 12/13 (p=0.36, OR=0.63, 95% CI, 0.25–1.55); neoplasia BK/FD=40/28 (p=0.68, OR=1.28, 95% CI, 0.57–2.88); respiratory disorders BK/FD=7/2 (p=0.19, OR=0.15, 95% CI, 0.01–3.12); trauma: only 2 cases in FD group. Donor gender distribution was similar in both groups: Male/Female ratio were 42/17 in BK and 23/22 in FD (p=0.04, OR=2.36, 95% CI, 1.05–5.32). The time lap between death and corneal tissue explant was similar for both groups (BK: 29 hours, FD: 21 hours; p=0.37, 95% CI, -1.28–3.43). There was no significant difference between the two groups in terms of donor corneas bank origin (Centro Conservazione Cornee “Piero Perelli” [Azienda USL n. 2, Ospedale Campo di Marte, Lucca, Italy] or Fondazione Banca degli Occhi del Veneto ONLUS [Zelarino (VE), Italy]). Lucca provided 29 corneas in BK and 21 in FD, while Mestre provided 30 corneas in BK and 24 in FD (p=0.84, OR=1.1, 95% CI, 0.51- 2.4). ARB was performed in 9 eyes affected by BK and in 19 affected by FD (p=0.003, OR= 0.25, 95% CI, 0.098–0.62). The mean time between surgery and ARB ± SD were 8±12.14 days (range: 0-32) for BK and 4.95±7.15 days (range: 1-32) for FD (p=0.41, 95% CI, -4.42 –10.53). Limiting our analysis to patients who underwent ARB showed that BK eyes received thicker tissues than FD ones. The mean donor corneal thickness ± SD were 778.89±196.04 µm (range: 547-1213) in BK and 665.89±61.34 µm (range: 573-786) in FD (p=0.03, 95% CI, -221.91–13.08). The mean± SD corneal decompensation period before surgery were 12.11±11.67 months (range: 1-36) in BK and 15.53±12.76 months (range: 4-60) in FD (p=0.5, 95% CI, -6.93–13.76). Aphakia was an exclusive concomitant eye disorder in BK eyes (p=0.02, OR=21, 95% CI, 0.95-463.4). In BK, ARB was associated to complicated cataract extraction (p=0.04, OR=7.83, 95% CI, 1.28–47.98) and aphakia (p=0.026, OR=54.38, 95% CI, 2.51-11.76) (Table 4). In FD, ARB was slightly associated to fatal disorder of donor patient, in particular to neoplasia (p=0.06, OR=4.04, 95% CI, 1.06–15.37) (Table 5).

## DISCUSSION

Statistical analysis of early GD rate in our study establishes the need of post-DSAEK ARB in about 28% of treated eyes, 16.4% of BK and 42.2% of FD. This finding confirms the literature data reporting mean GD rate as 14% [9, 10], or in a range of 4-27% [11, 14]. Focusing on BK group, our data confirmed a higher risk of GD in case of aphakia or in complicated cataract extraction. In literature the role of glaucoma in GD is controversial [15-18]. As recorded in recent studies by *Pavlovic et al.* [17], our data do not correlate GD to glaucoma both in BK and FD. Other investigated parameters (Tables 4, 5) did not correlate with GD in both groups, except for lenticules, explanted from patients affected by neoplasia, in FD where ARB was more frequent (42.2%) than in BK (15.2%). *Demsey* [19] demonstrated that graft dislocation is not influenced by variation in donor tissue processing and storage times. This evidence was extended to precut Eye Bank tissue thanks to *Dapena et al*. [14]. We excluded the influence on GD of time lapse between donor death and tissue implantation. A histopathological study of detached and failed graft conducted by *Alkatan et Al*., reported a higher risk of GD in case of irregular or thick graft, graft-host interface fibrous/epithelial ingrowth and interface infection [20]. Due to retrospective limitation of our study we could consider only the thickness of lenticules, as recorded by Eye Bank data forms. For both groups thickness of lenticules was similar, avoiding any interference related to tissue preparation. Moreover, ARB was successful and recovered GD in all cases, excluding any further postoperative analysis. We did not perform venting incision to prevent any postoperative complication in patients discharged the day after surgery. Even if venting incisions could improve the adherence of donor's lenticule [21], it might increase the risk of deep infectious keratitis [22] or induce corneal irregular astigmatism [23]. Mohebbi assumed that venting incision may not be necessary in the standard DSAEK procedures [24] we refrain to proceed with this technique. Anterior segment biomicroscopy did not reveal any sign of graft failure or graft rejection prior or after ARB [25, 26].

**Table 4:** Comparative Table Between Successful Implant and Air Rebubbling in Eyes Affected by Bullous Keratopathy Following Descemet's Stripping Automated Endothelial Keratoplasty.

	Air Re-Bubbling n or mean±SD (range)	Successful n or mean±SD (range)	Statistic p (95% CI)
Records	9	50	
Age(Y)	72.67±13.81 (51-87)	68.18±11.21 (38-87)	0.29 (-3.93 – 12.09)
M/F	2/7	27/23	0.15, OR 0.24 (0.05 – 1.29)
Patient Cornea			
Mean K (D) Max K (D) White to White (mm) Thickness (µm) Edema onset (months)	42.98±3.81 (33.99-46.92) 46±2.51 (42.19-49.93) 11.62±0.54 (10.6-12.4) 778.89±196.04 (547-1213) 12.11±11.67 (1-36)	43.58±2.54 (36.83-48.74) 45.4±2.83 (41.61-54.53) 11.63±0.53 (10.18-12.8) 848.82±191.4 (511-1388) 11.88±15.39 (1-84)	0.57 (-1.42 – 2.57) 0.55 (-2.62 – 1.42) 0.95 (-0.38 – 0.4) 0.32 (-69.32 – 209.19) 0.97 (-11.05 – 10.59)
Concomitant eye pathology Cornea Glaucoma Retinal disorder Complicated cataract Pseudophakia Aphakia	7 3 1 0 3 0 3	37 23 7 4 3 0 0	1, OR 1.23 (0.23 – 6.69) 0.72, OR 0.59 (0.13 – 2.61) 1, OR 0.77 (0.08 – 7.12) 1, OR 0.54 (0.03 – 10.97) **0.04**, OR 7.83 (1.28 – 47.98) - **0.026** OR: 54.38 (2.51 - 11.76.8)
Combined Surgery			
Phaco + IOL implant IOL explant + IOL implant	4 0	9 5	0.09 OR: 3.64 (0.81 – 16.33) 1 OR: 0.43 (0.02 – 85.56)
Donor Cornea			
Diameter of cut tissue (mm) Thickness (µm) Endothelial cell count (/mm^2^) Diameter of implanted tissue (mm)	10.19±0.97 (9-11) 95±28.97 (45-135) 2577.78±120.19 (2400-2700) 8.±0.25 (7.5-8.25)	9.81±0.81 (9-11) 100.26±37.68 (43-285) 2614.26±110.68 (2300-2800) 8.06±0.36 (6.5-8.5)	0.22 (-0.98 – 0.23) 0.69 (-21.27 – 31.78) 0.38 (-45.03 – 117.47) 0.63 (-0.19 – 0.31)
Donor Patient			
Age(Y) M/F	70.78±6 (60-78) 7/2	64.76±8.79 (42-79) 35/15	0.05 (-12.15 – 0.11) 1 OR: 1.5 (0.28 – 8.08)
Death pathology			
Vascular Disorders Cancer Respiratory Disorders Trauma Time of death to explant (hours) Lucca (L) Eye Bank (tissues) Mestre (M) Eye Bank (tissues)	2 6 1 0 8.28±5.9 (3-21.5) 4 5	10 34 6 0 9.19±5.56 (2.5-23.5) 25 25	1 OR: 1.14 (0.2 – 6.37) 1 OR: 0.94 (0.21 – 4.25) 1 OR: 0.91 (0.1 – 8.67) - 0.65 (-3.15 – 4.98) L versus M: 1 OR: 0.8 (0.19 - 3.33)
Sex Match			
Pt M –donor M Pt F –donor F Pt M – donor F Pt F – donor M	1 1 1 6	19 7 8 16	0.5 OR: 0.37 (0.02 – 6.73) 0.64 OR: 0.33 (0.03 – 3.26)

Abbreviations: n: number; SD: Standard Deviation; CI: Confidence Interval; P: p-value; Y: year; M: male; F: female; Max: maximum; K: keratometry reading; D: diopter; mm: millimeter; µm: micrometer; /mm2: cells per millimeter square; OR: odds ratio; Phaco: phacoemulsification; IOL: intraocular lens; Pt: patient.

**Table 5:** Comparative Table Between Successful Implant and Air Rebubbling in Eyes Affected by Fuchs Dystrophy Following Descemet's Stripping Automated Endothelial Keratoplasty.

	Air Re-Bubbling n or mean±SD (range)	Successful n or mean±SD (range)	Statistic p (95% CI)
Records	19	27	
Age(Y)	73.32±7.76 (55-85)	68.46±9.56 (47-86)	**0.07** (-10.24 – 0.53)
M/F	8/11	16/7	0. 21, OR 0.32 (0.09 – 1.14)
Patient Cornea			
Mean K (D) Max K (D) White to White (mm) Thickness (µm) Edema onset (months)	43.85±1.81 (40.86-46.5) 44.80±1.89 (41.41-47.3) 11.46±0.36 (10.5-12.12) 665.89±61.34 (573-786) 15.53±12.76 (4-60)	44.13±2.76 (41.26-55.86) 45.05±30.3 (41.54-58.24) 11.69±0.47 (10.8-12.9) 709.77±117.82 (545-2101) 13.08±13.92 (2-72)	0.7 (-1.18 – 1.75) 0.76 (-1.35 – 1.83) 0.08 (-0.03 – 0.49) 0.14 (-15.9 – 103.65) 0.55 (-10.64 – 5.74)
Concomitant eye pathology Cornea Glaucoma Retinal disorder Complicated cataract Pseudophakia Aphakia	3 2 0 1 0 0 0	5 1 1 3 0 0 0	1, OR 0.82 (0.17 – 3.96) 0.56, OR 3.06 (0.26 – 36.44) 1, OR 0.45 (0.02 – 11.73) 0.63, OR 0.44 (0.04 – 4.63) - - -
Combined Surgery			
Phaco + IOL implant IOL explant + IOL implant	13 0	24 0	0.13 OR: 0.27 (0.06 – 1.27)
Donor Cornea			
Diameter of cut tissue (mm) Thickness (µm) Endothelial cell count (/mm^2^) Diameter of implanted tissue (mm)	9.86±0.9 (9-11) 105.32±26.42 (62-149) 2605.26±131.12 (2300-2800) 8.09±0.15 (7.75-8.25)	9.78±0.79 (9-11) 104.04±21.39 (68-154) 2603.85±111.29 (2400-2800) 8.05±0.22 (7.5-8.5)	0.75 (-0.59 – 0.43) 0.85 (-15.66 – 13.1) 0.97 (-74.45 – 71.61) 0.46 (-0.16 – 0.07)
Donor Patient			
Age (Y) M/F	63.74±9.37 (39-74) 9/10	60.19±10.8 (36-79) 14/13	0.26 (-9.77 – 2.68) 1 OR: 0.84 (0.26 – 2.71)
Death pathology			
Vascular Disorders Cancer Respiratory Disorders Trauma Time of death to explant (hours) Lucca (L) Eye Bank (tissues) Mestre (M) Eye Bank (tissues)	4 15 0 0 8.26±6 (2.5-22.5) 10 9	9 13 2 2 11.48±6.66 (2.5-3) 11 16	0.51 OR: 0.53 (0.14 – 2.08) **0.06** OR: 4.04 (1.06 – 15.37) 0.5 OR: 0.26 (0.02 – 5.77) 0.5 OR: 0.26 (0.02 – 5.77) 0.1 (-0.67 – 7.1) L versus M: 0.55 OR: 0.62 (0.19 - 2.02)
Sex Match			
Pt M –donor M Pt F –donor F Pt M – donor F Pt F – donor M	3 5 5 6	8 4 8 6	0.36 OR: 0.3 (0.05 – 1.9) - - 0.7 OR: 0.62 (0.13 – 30.7)

Abbreviations: n: number; SD: Standard Deviation; CI: Confidence Interval; P: p-value; Y: year; M: male; F: female; Max: maximum; K: keratometry reading; D: diopter; mm: millimeter; µm: micrometer; /mm2: cells per millimeter square; OR: odds ratio; Phaco: phacoemulsification; IOL: intraocular lens; Pt: patient. P-value<0.05 is bold.

The exclusion of those risk factors in FD enhanced the correlation between lenticules explanted from patients affected by neoplasia and GD. This evidence points toward a causative relationship between the FD physiopathology and GD. Assuming that we did not find historical evidence of congenital corneal disorder, FD is an autosomal dominant disease that affects deeper corneal layers collagen [27] that are partially stripped in DSAEK and partially prepared to attach to graft. DSAEK technique realizes the stripping of the Descemet-Endothelium complex. Probably the deeper stroma next to Descemet’s membrane is the weak link in graft adhesion, due to its anatomical and functional damage [28, 29]. Many reports have studied the effect of chemotherapy for different type of cancer on systemic tissues, especially on connective layers, inducing jeopardized disorders [30-33]. Along this evidence we suppose that chemotherapy affects donor’s corneal stroma, interfering with fibroblastic metabolism [34]. This feature is not relevant in BK but could be critical on corneas affected by congenital and metabolic disorders as in FD, increasing the risk of GD [3, 35, 36]. Due to our limited access to donors’ clinical history, we do not have any data about neither donors’ neoplasia nor related chemotherapy. 

Strengths of the study included homogeneous sample and experienced single-surgeon (G.A.). Our data are limited by the retrospective design of the study. We considered ARB and anatomical recovery of GD in BK and FD. Histopathologic examination of donor’s detached lenticule was not performed because in all eyes GD was totally recovered by ARB. Long-term postoperative functional and anatomical evaluation was not recorded because patients were discharged immediately after surgery and followed by territorial ophthalmic offices. Therefore, future studies by eliminating these limitations could be more informative and valuable.

## CONCLUSIONS

Although this study showed a complete and easy graft re-attachment with ARB, it might expose patients to further risks. A desirable outcome would be identification of the risk factors of GD to limit further surgical approaches. We found that, as widely reported in the literature, aphakia and complicated cataract extraction increase the risk of GD in BK. To our knowledge for the first time we identified that graft explanted from neoplastic donor may impair DSAEK in FD, increasing GD rate of 2.6. 
